# Gastroprotective Effect of Ethanol Extracts from Bark of Magnolia officinalis on Ethanol-Induced Gastric Mucosal Damage in Rats

**DOI:** 10.1155/2021/6688414

**Published:** 2021-05-31

**Authors:** Xiao Wang, Shu Fu, Chen Zhang, Xin Nie, Wan Liao, Ming Zhao, Fang Liu

**Affiliations:** ^1^Pharmacy College, Chengdu University of Traditional Chinese Medicine, Chengdu 611137, China; ^2^Ya'an Food and Drug Inspection Institute, Ya'an, 625000 Sichuan, China

## Abstract

*Background. Magnolia officinalis* Rehd. and Wils. is widely used in Asian countries because of its multiple pharmacological effects. This study investigated the gastroprotective effect and mechanisms of the ethanol extracts from the bark of Magnolia officinalis (MOE) against ethanol-induced gastric mucosal damage in rats. *Methods.* MOE was prepared by reflux extraction with 70% ethanol, and its main compounds were analyzed by UPLC-Q-Exactive Orbitrap-MS. DPPH, ABTS, and FRAP methods were used to evaluate the antioxidant capacity of MOE in *vitro*. The gastroprotective effects of MOE were evaluated by the area of gastric injury, H&E (hematoxylin-eosin), and PAS (periodic acid-Schiff). The mechanism was explored by measuring the levels of cytokines and protein in the NF-*κ*B signaling pathway. *Results.* 30 compounds were identified from MOE, mainly including lignans and alkaloids. MOE presented a high antioxidant activity in several oxidant *in vitro* systems. Gastric ulcer index and histological examination showed that MOE reduced ethanol-induced gastric mucosal injury in a dose-dependent manner. MOE pretreatment significantly restored the depleted activity of superoxide dismutase (SOD), glutathione peroxidase (GSH-Px) enzymes, reduced malondialdehyde (MDA), and prostaglandin E2 (PGE2) levels in the gastric tissue in rats. In addition, MOE also inhibited the activation of nuclear factor kappa B (NF-*κ*B) pathway and decreased the production of proinflammatory cytokines. *Conclusions.* The gastroprotective effect of MOE was attributed to the inhibition of oxidative stress and the NF-*κ*B inflammatory pathway. The results provided substantial evidence that MOE could be a promising phytomedicine for gastric ulcer prevention.

## 1. Background

Gastric ulcer is a major disease of the gastrointestinal tract system, which causes harm to 10% of the world population in varying degrees [1]. Its high incidence rate, serious complications, and various inducements cause adverse effects on human health [2]. Gastric ulcer can be induced by *Helicobacter pylori* infection, stress, smoking, excessive intake of nonsteroidal anti-inflammatory drugs, and excessive alcohol [3, 4]. Alcohol is the most common damage factor in daily life, which usually leads to gastric tissue lesions, such as gastritis, gastric ulcer, and even gastric cancer [5]. The mechanism of ethanol-induced gastric injury has not been fully elucidated, but increasing evidence has shown that ethanol can directly damage gastric mucosa through destruction, dehydration, and mucosal cytotoxicity. At the same time, ethanol-induced inflammation, oxidative stress, and apoptosis through leukocyte recruitment, which further indirectly damaged gastric mucosa [6]. Chemicals are commonly used to treat gastric ulcer, such as antibiotics, proton pump inhibitors, antacids, and antihistamines [7]. However, they are associated with a number of side effects, such as vitamin B12 deficiency, depression, and headache, which suggests that it is necessary to look for natural medicines as alternatives to treat gastric ulcer [2, 8].

*Magnolia officinalis* var. biloba Rehder and Wilson is a famous bulk medicinal material in Asia. Its medicinal part is the stem bark, which is traditionally used to treat gastrointestinal diseases, anxiety, asthma, headache, and other diseases [9]. Magnolia officinalis is often extracted and prepared into a variety of Chinese patent medicines for the treatment of gastrointestinal diseases, such as Huoxiang Zhengqi water, Pingwei powder, and Zhishi Xiaopi pill. Magnolol and honokiol, the main active components of bark extract of Magnolia officinalis, are phenolic compounds that can inhibit the proton pump of gastric mucosal parietal cells and reduce the production of acidic gastric juice and lipid peroxidation [10]. Moreover, they can also affect inflammation-related indicators, such as IL-1*β*, TNF *α*, and COX-2 [9, 11]. Magnolol and honokiol in Magnolia officinalis extract have antioxidation and anti-inflammatory pharmacological effects [12], corresponding to the pathogenesis of ethanol-induced gastric ulcer. Therefore, we speculate that Magnolia officinalis extract can repair gastric mucosal injury induced by ethanol, and it is a potential natural product for the treatment of gastric ulcer. However, the composition of the Magnolia officinalis extract (MOE) is complex, and its protective mechanism on ethanol-induced gastric mucosal injury is not fully understood.

In this study, we analyzed the main components of the Magnolia officinalis extract by UPLC-Q-Exactive Orbitrap-MS and investigated the mechanism of MOE improving gastric ulcer induced by ethanol from the aspects of inhibiting oxidative stress and anti-inflammatory, so as to provide the basis for the preparation development and clinical application of MOE.

## 2. Methods

### 2.1. Reagents and Material

The kits for the biochemical analysis of superoxide dismutase (SOD), MDA, and GSH-Px were purchased from Jiancheng Bioengineering Institute (Nanjing, China). The enzyme-linked immunosorbent assay (ELISA) kits for TNF-*α*, IL-1*β*, IL-6, and PGE2 and the antibodies against COX-2, NF-*κ*B p65, p-NF-*κ*B p65, IKB*α*, and p-IKB*α* were supplied by MultiScience (Lianke) Biotech Co., Ltd. (Hangzhou, China). Magnolol and honokiol were purchased from Chengdu RuiFenSi Bio-Tech Co., Ltd. HPLC-grade acetonitrile and water used in this study were purchased from Fisher Scientific-UK. All other regents were of analytical grade.

### 2.2. Plant Collection and Extraction

The bark of *Magnolia officinalis* Rehd. and Wils. was collected in Hongkou Township, Dujiangyan City, Sichuan Province (N 31°07′0.56^″^, E 103°40′1.81^″^) in June 2018, which is a famous production origin of Magnolia officinalis. A representative sample of the species was identified by Yan Lian from Chengdu University of Traditional Chinese Medicine, Chengdu, China.

The barks from Magnolia officinalis were weighed and crushed, followed by extraction by reflux method with 70% ethanol for three times, 90 mins each time. The ethanol extract was filtered to collect the filtrate. The filtrate was concentrated on a rotary evaporator with a temperature of about 50°C and 40 rpm and then freeze-dried to obtain MOE. Before the experiment, MOE was stored in a desiccator for standby.

### 2.3. UPLC-Q-Exactive Orbitrap-MS Analysis

The chemical composition of MOE was determined by UPLC-Q-Exactive Orbitrap-MS mass spectrometer (USA, Thermo Fisher Company). Chromatographic separation was performed on a reverse phase Sunfire C18 column (3.0 mm × 150 mm, 3.5 *μ*m). Mobile phase composed of water (A) and acetonitrile (B). The program of gradient elution was 90% acetonitrile at 0–15 min, 90–25% acetonitrile at 15–25 min, 25–25% acetonitrile at 25–35 min, and 5% acetonitrile at 35–40 min. The flow rate and the injection volume were 0.2 mL/min and 2 *μ*L, respectively. Column temperature was 35°C, and PDA detection was at 270 nm. Standards used to identify compounds were magnolol (MB2181-S), honokiol (MB5989-S), magnoflorine (MB4437), and syringin (MB7084).

The mass spectrometric condition is as follows: ion source, electrospray ion source (ESI) mixed ion source mode; *m*/*z* detection range, 150~1000; detection mode, positive/negative ion; collision voltage, 35 V; capillary voltage 3.2 kV; capillary temperature, 320°C; and dry air temperature: 350°C.

### 2.4. Determination of Antioxidant Activity In Vitro

The antioxidant activity (DPPH, ABTS, and FRAP) of MOE was measured following the methodology published by Cesario et al. [13] and Can-Cauich et al. [14]. For each test, a calibration curve was prepared using ascorbic acid as the standard.

Compared with the control group (PBS solution), the free radical inhibition rate of the sample was calculated as follows.

The percentage of inhibition in comparison to the control was calculated as follows:

(1) $$\begin{array}{c} \frac{\text{Inhibition of DPPH}}{\text{ABTS}}\left(\%\right)=\left[\frac{\left(\text{Abs control}-\text{Abs sample}\right)}{\text{Abs control}}\right]\times 100\%. \end{array}$$

Abs sample was the absorbance of the sample; Abs control was the absorbance of the control. Using the calibration curve of DPPH/ABTS inhibition rate (%) at different concentrations, the sample volume required to reduce the initial DPPH/ABTS concentration by 50% (EC50) was calculated, expressed in mg/mL.

### 2.5. Gastroprotective Effects of MOE in Rats

#### 2.5.1. Animals

Male Sprague-Dawley (SD) rats weighing 200-250 g were obtained from Chengdu Dossy Experimental Animal Co. Ltd. (Permit No. SCXK (Chuan) 2015-30, Chengdu, China). The animals were maintained under controlled conditions at temperature 20 ± 0.5°C, humidity 55 ± 5%, and with 12 h light and 12 h dark cycles. All rats were acclimatized for 7 days before any experiments and were fed with standard chow and water ad libitum. Animal experiments were conducted in strict accordance with the recommendations of the Guidelines for the Care and Use of Laboratory Animals of the Ministry of Science and Technology of China. The protocol and experimental designs were approved by the Ethical Committee of Chengdu University of Traditional Chinese Medicine. At the end of study, the animals were sacrificed following anesthesia with pentobarbital sodium (100 mg/kg).

#### 2.5.2. Modeling and Administration

All animals were randomly divided into six groups consisting of six animals in each. The rats in the control and model groups were treated with 0.5% CMC-Na (10 mL/kg, p.o.). The rats in the positive group were treated with omeprazole dissolved in 0.5% CMC-Na (30 mg/kg, p.o.). And the rats in MOE groups were treated with different doses of MOE in 0.5% CMC-Na (30, 60 or 120 mg/kg, p.o.) [15]. The dosage of Magnolia officinalis in the Chinese pharmacopoeia for human is 3~10 g/d [16]. The dosage of extract should be converted according to the principle of dosage conversion between the human and rats; combined with the yield of the extract, the dose of MOE is 33~110 mg/kg [17]. According to the theoretical conversion value, the low, middle, and high doses of MOE in rats were set as 30, 60, and 120 mg/kg, respectively. The corresponding medicine oral administration in rat was once per day for 4 days. The rats fasted 24 hours before death but drank freely. 2 h after the last administration, except for the normal control group, all rats were given 5 mL/kg ethanol to induce gastric mucosal injury [18, 19]. 1 h later, the rats were anesthetized to death with pentobarbital sodium. Then, their abdominal cavity was opened, and stomachs were taken out and cleaned with PBS. Dry the cleaned stomach with filter paper and collect the whole stomach image by a camera (Nikon Inc., Japan) immediately. After that, the stomach was dissected into two parts longitudinally: one was fixed with 10% formaldehyde for pathological section, and the other was temporarily stored at -80°C for further study.

Image-Pro Plus software (Media Cybernetics, USA) was used to process the image and calculate the ratio between the area of hemorrhagic ulcer and the total gastric mucosa [20]. The gastric tissue fixed with 10% formalin was embedded in paraffin and cut into 5*μ*m sections and stained with hematoxylin, eosin, and periodic acid-Schiff, respectively [21]. In brief, the slides containing tissue sections were dewaxed in xylene and rehydrated in a series of ethanol; then, periodic acid solutions were added to the slides and incubated for 5min. The Schiff's reagent was added onto the slides for 15 min following rinsing off the periodic acid. The nuclei were counterstained in hematoxylin.

#### 2.5.3. Measurement of SOD, MDA, GSH-Px, and PGE2 Levels

A part of the gastric tissue stored at -80°C was homogenized with normal saline and centrifuged at 1200 rpm for 10 minutes at 4°C. The BCA method was used to determine the protein content in gastric samples. The contents of SOD, MDA, GSH-Px, and PGE2 in gastric tissue were determined by a kit. All operation steps shall be carried out according to the instructions on the kit.

#### 2.5.4. Evaluation of Cytokines in Gastric Tissues

The content of TNF-*α*, IL-1*β*, and IL-6 in the supernatant of gastric tissue by enzyme-linked immunosorbent assay kits according to the manufacturer's specifications [22]. The absorbance was measured at 450 nm.

#### 2.5.5. Western Blot Analysis of Stomach Tissue

Another part of the stomach tissue stored at -80°C is added with protease inhibitor and homogenized with cold normal saline and centrifuged at 12000 rpm at 4°C for 10 min, and the supernatant was taken for standby. After the preparation of polyacrylamide gel, gel perfusion, sample addition, electrophoresis, transfer membrane, chemical reaction, and immobilization, the experimental images were obtained. Finally, the alpha software processing system was used to process the film image and analyzed the optical density of the target band. The contents of COX-2, NF-*κ*B p65, p-NF-*κ*B p65, IKB*α*, and p-IKB*α* were all determined by this way.

#### 2.5.6. Statistical Analysis

All data were expressed as mean ± standard deviation (SD) and analyzed with GraphPad Prism 5.0 (GraphPad Software, San Diego, USA). The one-way analysis of variance (ANOVA) test was used to compare the data. The value of *p* < 0.05 was considered as a significant difference.

## 3. Results

### 3.1. The UPLC-Q-Exactive Orbitrap-MS Analysis of MOE

**Figure 1:**
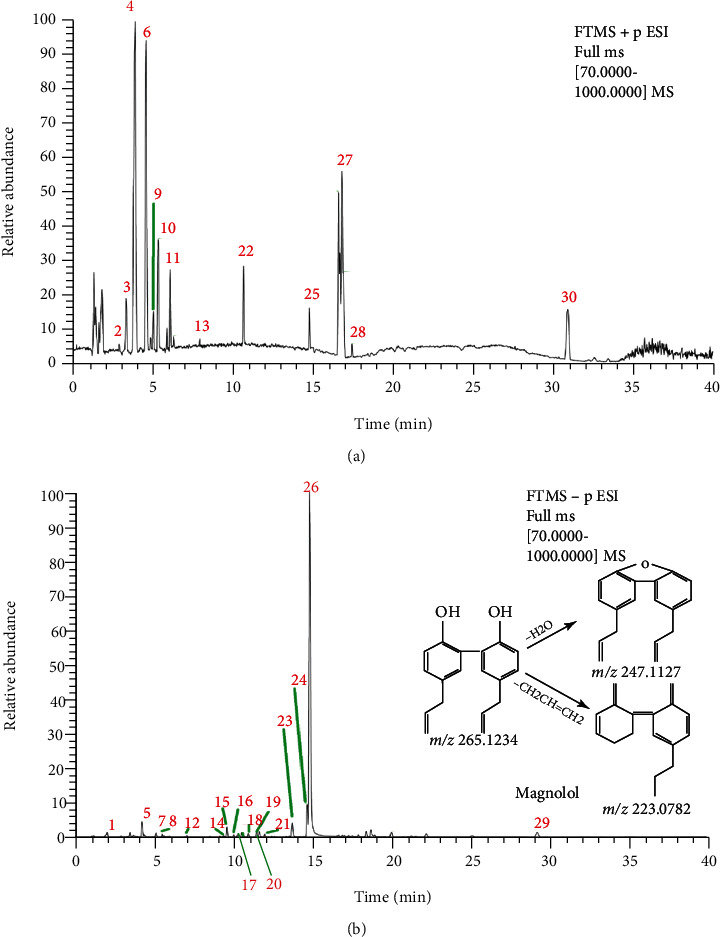
UPLC-Q-Exactive Orbitrap-MS analysis of MOE. (a) Total ion chromatogram in positive ion mode). (b) Total ion chromatogram in negative ion mode.

The collection of barks from Magnolia officinalis had a weighing of 250 g. After pretreatment and ethanol extraction, 30.98 g extract was obtained, so the yield of MOE was 12.39%. Chemical analysis was performed using UPLC-Q-Exactive Orbitrap-MS and total ion current chromatogram in positive and negative ESI modes is shown in Figures 1(a) and 1(b). The MS_2_ chromatograms and the proposed fragmentation mode of magnolol, the main active components in MOE, are shown in Figure 1(d). In both positive and negative modes, the components in MOE were completely separated. The original data were preliminarily analyzed and processed by Compound Discoverer software. Compounds with a matching degree of more than 80 points and delta mass range less than ±5 ppm were screened. Referring to the relevant literature of Magnolia officinalis, 30 provisional compounds were screened; the results are shown in Table 1 [23].

### 3.2. The Antioxidant Activity of MOE In Vitro

**Figure 2:**
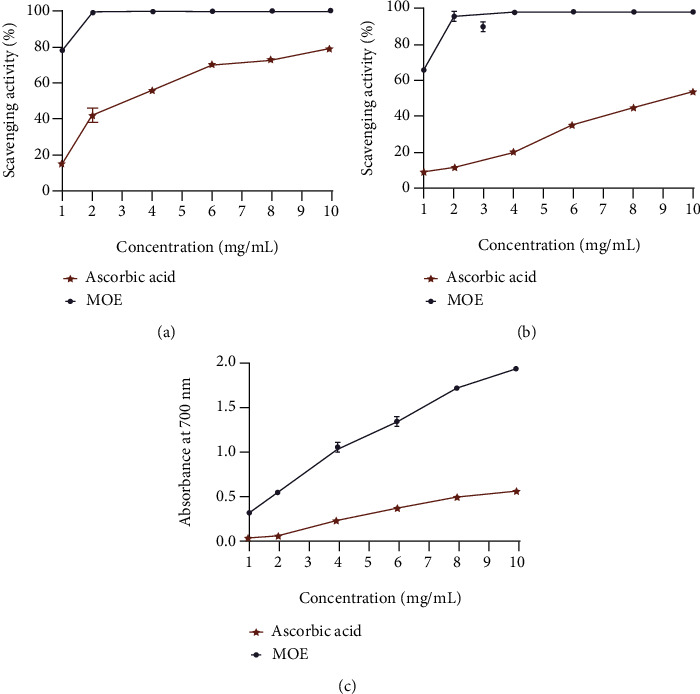
*In vitro* antioxidant activity of MOE. (a) Antioxidant activity of MOE by OPPH scavenging assay. (b) Antioxidant activity of MOE by ABTS scavenging assay. (c) Antioxidant activity of MOE by FRAP.

Oxidative stress is believed to initiate and aggravate many digestive system diseases, including gastric ulcers. Antioxidants play a major role in counteracting excessive free radical generation that may occur during ulcer formation by scavenging free radical formation [13, 24, 25]. The antioxidant activity of the extracts was evaluated in vitro, and the results are presented in Figure 2. It showed that the radical scavenging activity of MOE and ascorbic acid on DPPH, ABTS, and FRAP radicals increased in a dose-dependent manner. The IC50 of MOE for DPPH radical scavenging was 2.13 ± 0.05 mg/mL (Figure 2(a)). In addition, the IC50 for MOE was 14.73 ± 0.02 mg/mL in the analysis of the chelating activity of ABTS free radicals (Figure 2(b)). The determination of iron ion reduction ability showed the electron donating ability of antioxidants. The results showed that the reduction ability of MOE and ascorbic acid increased with the increase of concentration (Figure 2(c)). The antioxidant capacity of the three indexes measured showed that MOE has a strong antioxidant activity *in vitro* which was the basis of inhibiting oxidative stress.

### 3.3. MOE Alleviated Ethanol-Induced Gastric Injury

**Figure 3:**
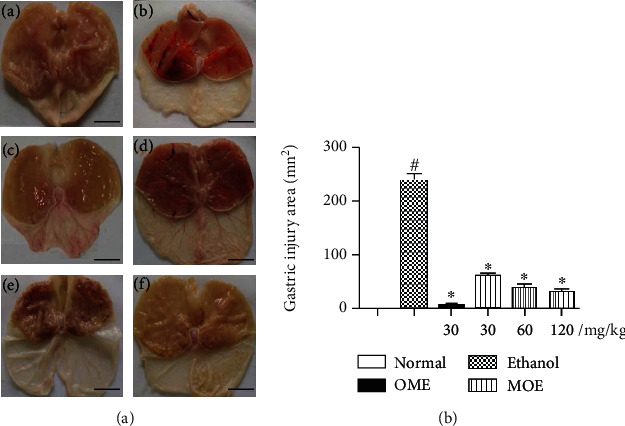
Effects of MOE pretreatment on the macroscopic appearance of the gastric mucosa in ethanol-induced gastric injury rats (*n* = 6). (a) Photos of gastric mucosal injury ((A) normal group, (B) ethanol group, (C) omeprazole group, (D) 30mg/kg of the MOE group, (E) 60 mg/kg of the MOE group, and (F) 120mg/kg of the MOE group). (b) Quantitative analysis of the gastric injury area was assessed by Image-Pro Plus software. Data were expressed as mean ± SDs. ^#^*p* < 0.05, compared with the normal group, ^∗^*p* < 0.05 compared with the ethanol group.

After oral administration of excessive alcohol, gastric mucosal edema, hyperemia of glandular region, and linear bleeding were observed [26, 27]. The degree of gastric mucosal injury induced by ethanol and the effect of medicines improvement are shown in Figure 3. In the normal group, the gastric mucosa was smooth and intact without damage. In the ethanol group, obvious mucosal edema and linear bleeding were observed, which indicated that gastric ulcer model could be successfully induced by ethanol gavage (Figure 3(a)). In the low-dose group (30 mg/kg) of MOE, there were a small amount of bleeding points in the gastric tissue and congestion in the glandular area. In the medium-dose group (60 mg/kg), there were only a few bleeding points in the gastric tissue, and the congestion in the glandular area had been basically improved. In the high-dose group (120 mg/kg), the gastric mucosa recovered completely without bleeding points. The treatment groups showed that MOE reduced bleeding and swelling in a dose-dependent manner. In the positive control rats treated with omeprazole, the symptoms of gastric mucosal hemorrhage and edema were completely improved.

The area of gastric injury was used as an index to investigate the therapeutic effect of MOE on ethanol induced gastric injury. As shown in Figure 3(b), the area of gastric mucosal injury in the model group was significantly higher than that in normal group (*p* < 0.05). Oral administration of omeprazole or MOE significantly reduced the mucosal injury area induced by ethanol (*p* < 0.05). The average area of gastric injury in the model group was 239.18 ± 32.15 mm^2^. Omeprazole treatment exhibited 97% reduction in the ulcer area. Meanwhile, after pretreatment of 30 mg/kg of MOE, almost 74% decrease of ulcer area was found as compared to the ulcer model group, 60 mg/kg of MOE treatment exhibited 84% reduction in the ulcer area, and 120 mg/kg of MOE treatment exhibited 87% reduction in the ulcer area. These results indicated that MOE had a significant effect on the repair of ethanol-induced gastric injury in a dose-dependent manner.

### 3.4. Histological Evaluation: H&E and PAS Staining of Gastric Lesions

**Figure 4:**
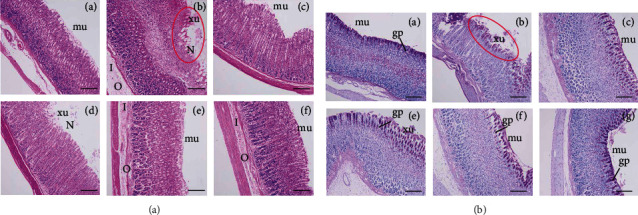
Effects of MOE on the microscopic aspect of the gastric mucosa in ethanol-induced gastric mucosal damaged rats (*n* = 6). (magnification 100x, Scale bar: 50 *μ*m). (a) H&E staining. (b) PAS staining ((A) normal control group, (B) ethanol group, (C) omeprazole group, (D) 30 mg/kg of the MOE group, (E) 60 mg/kg of the MOE group, and (F) 120 mg/kg of the MOE group. N: necrosis. O: edema, I: inflammatory cells infiltration in submucosa, mu: intact mucosal layer, xu: focal exulceration; gp: glycoprotein).

The results of H&E staining showed that ethanol could damage the gastric mucosa and cause gastric ulcer. In the normal group, the mucosal layer on the surface of gastric tissue was not damaged, and the epithelial cells were intact without bleeding and inflammatory infiltration. Compared with the normal group, the gastric mucosa of the model group was completely destroyed, epithelial cells fell off, accompanied by significant bleeding points, and inflammatory cell infiltration was more serious (Figure 4(a)). In the low-dose group of MOE (30 mg/kg), the mucosal layer was destroyed and a few epithelial cells fell off. In the middle-dose group (60 mg/kg) and high-dose group (120 mg/kg) of MOE, complete epithelial tissue, no obvious bleeding point, and a small amount of inflammatory cell infiltration were observed. The protective effect of MOE on the gastric tissue was observed more finely from the tissue section.

In addition, periodic acid-Schiff (PAS) staining was used to detect the distribution of glycoproteins in gastric epithelial cells. The mucus secreted by the gastrointestinal tract is an important part of the mucosal defense. The acidic mucopolysaccharide contained in the mucus is the main protective layer of gastric epithelium, and the integrity of the acid mucopolysaccharide can reflect the degree of gastric injury [20]. Compared with the normal group, the mucopolysaccharide layer of gastric epithelium in the ethanol group was seriously damaged, and the mucopolysaccharide almost completely fell off (Figure 4(b)). Compared with the ethanol group, the mucopolysaccharide layer in the omeprazole group was more complete, but the distribution was more scattered. Similarly, the three MOE treatment groups retained intact mucopolysaccharide layer, and with the increase of concentration, the mucopolysaccharide layer became more compact. These results suggest that MOE can protect the stomach by maintaining the integrity of gastric mucosa.

### 3.5. MOE Reduced Oxidative Stress in the Damaged Gastric Tissue

**Figure 5:**
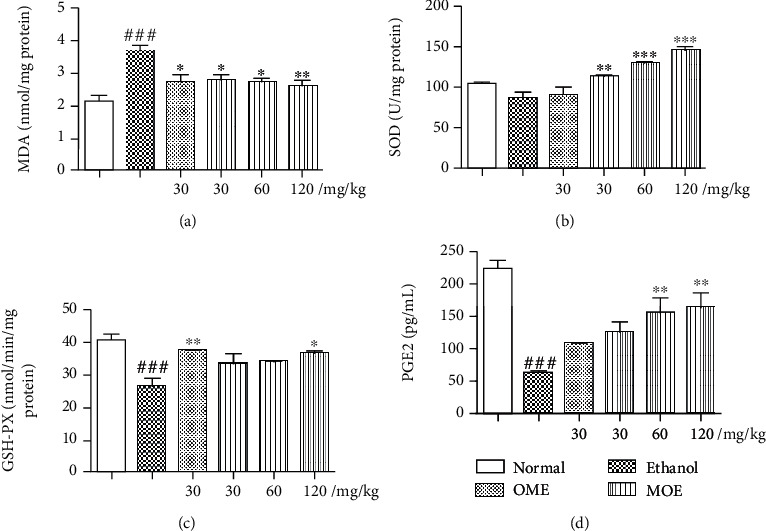
MOE inhibited oxidative stress response in the ethanol-induced gastric mucosal damaged rats. (a) MDA. (b) SOD. (c) GSH-Px. (d). PGE2. Data are expressed as the mean ± SD (*n* = 6). ^###^*p* < 0.001 compared with the normal group; ^∗^*p* < 0.05, ^∗∗^*p* < 0.01, and ^∗∗∗^*p* < 0.001 compared with the ethanol group.

The gastric mucosa of rats with ethanol induced gastric injury showed obvious disorder of oxidative stress markers, as shown in Figure 5. Compared with the normal group, the oxidative stress markers of the ethanol group were significantly different, and the MDA activity of the model group increased by 72%, while the SOD and GSH-Px activity decreased by 18% and 37%, respectively. The omeprazole group and three MOE treatment groups could reduce the production of MDA and increase the activities of SOD and GSH-Px. Among them, the high-dose MOE group (120 mg/kg) had a significant effect on the three markers of oxidative stress (*p* < 0.01).

The gastric mucosal PGE2 concentration was considerably reduced in the ethanol-induced gastric injury model to approximately 27% of the normal level (*p* < 0.001). MOE (60 and 120 mg/kg) pretreatment significantly increased the elevated PGE2 level by 149% and 166%, respectively (Figure 5(d), *p* < 0.01) compared with the model group. These results confirmed that MOE can reduce oxidative stress induced by excessive ethanol.

### 3.6. MOE Reduced Inflammatory Response in the Damaged Gastric Tissue

**Figure 6:**
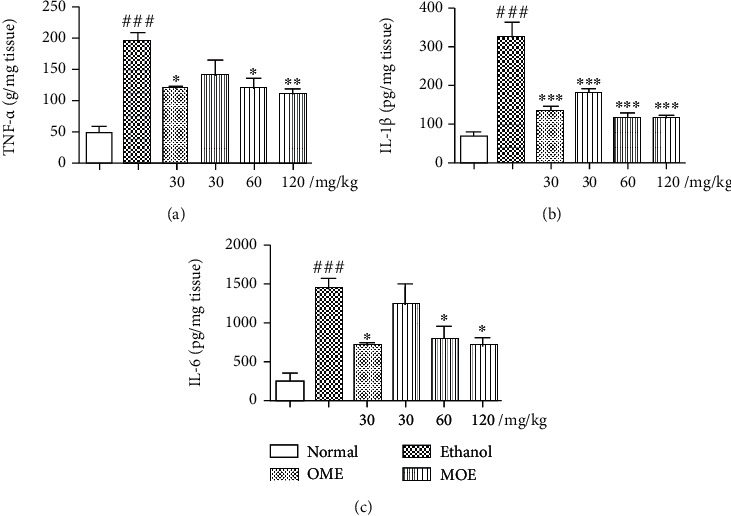
MOE inhibited ethanol-induced gastric mucosal inflammatory responses in ethanol-induced gastric mucosal damaged rats. (a) TNF-*α*. (b) IL-1*β*. (c) IL-6. Data are expressed as the mean ± SD (n =6). ^###^*p* < 0.001 compared with the normal group; ^∗^*p* < 0.05, ^∗∗^*p* < 0.01, and ^∗∗∗^*p* < 0.01 compared with the ethanol-induced gastric injury model group.

Excessive inflammatory factors are expressed in the gastric tissue of rats with gastric injury induced by ethanol, and the results are shown in Figure 6 Compared with normal rats, the contents of TNF-*α*, IL-1*β*, and IL-6 in the ethanol group were significantly increased (*p* < 0.05). Compared with the ethanol group, the omeprazole group significantly reduced the levels of these three inflammatory cytokines (*p* < 0.01). Pretreatment with MOE were able to reduce the TNF-*α*, IL-1*β*, and IL-6 concentrations compared with the ethanol group in dose-dependent manners.

### 3.7. MOE Blocked Ethanol-Induced Activation of NF-*κ*B Signaling Pathways

**Figure 7:**
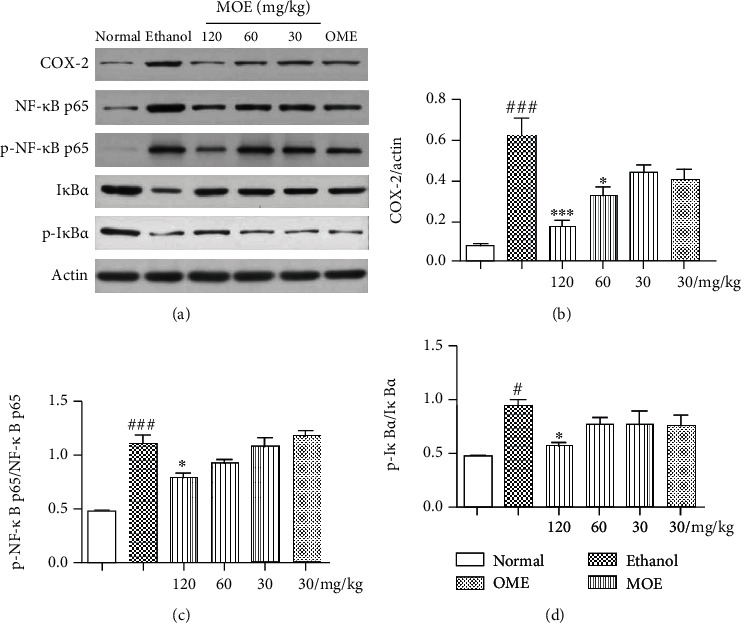
MOE blockaded NF-*κ*B pathways in ethanol-induced gastric mucosal damaged rats. Each bar represents the mean ± SD, *n* = 3. ^###^*p* < 0.001 compared with the normal group; ^#^*p* < 0.05 compared with the normal group; ^∗∗∗^*p* < 0.001 with the ethanol-induced gastric injury model group; ^∗^*p* < 0.05 with the ethanol-induced gastric injury model group.

In view of the abovementioned inflammatory response to alcoholic injury, we further explore the regulatory effect of MOE on the NF-*κ*B signaling pathway, which is commonly involved in the inflammatory signaling cascades. Ethanol activated the NF-*κ*B signaling pathway in the gastric tissue of rats, resulting in a significant increase in the expression of p-NF-*κ*B p65/NF-*κ*B p65, p-I*κ*B*α*/I*κ*B*α*, and COX-2 protein (Figure 7). Fortunately, the activation of NF-*κ*B signal transduction was reduced in all three MOE groups, and there was a significant difference in the high-dose MOE group (*p* < 0.05). Omeprazole is a proton pump inhibitor, which can inhibit the activity of H^+^-K^+^-ATPase and gastric acid secretion. It can also reduce the activity of pepsinase and promote the repair of the gastric mucosa [15]. However, omeprazole had no significant effect on the NF-*κ*B pathway, because compared with the model group, there was no significant difference in the contents of p-NF-*κ*B p65/NF-*κ*B p65, p-I*κ*B*α*/I*κ*B*α*, and COX-2. The results showed that MOE could inhibit the activation of the NF-*κ*B pathway.

## 4. Discussion

**Figure 8:**
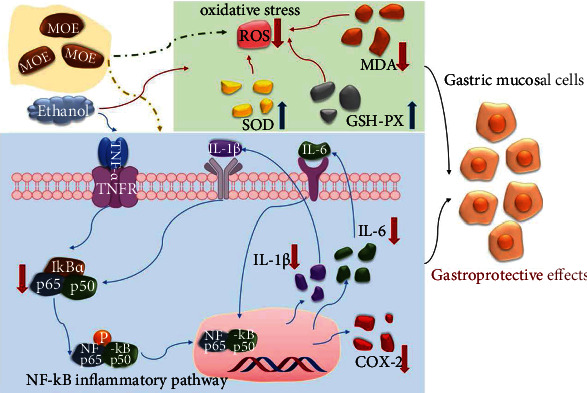
Mechanism of the gastroprotective effects of MOE on ethanol-induced gastric mucosal injury in rats.

Research on medicinal plants for disease treatment and prevention exists around the world, for compounds extracted from plants have great potential for disease prevention and treatment [28, 29]. *Magnolia officinalis* has a long medicinal history, and its research is also deepening [30]. Literature search showed that *Magnolia officinalis* mainly contains phenols, alkaloids, volatile oils, and other components [23]. In traditional Chinese medicine, Magnolia officinalis is often used to treat gastrointestinal diseases. Modern research also found that Magnolia officinalis has an inhibitory effect on *Helicobacter pylori* [31], so it has a therapeutic effect on gastric ulcer caused by *Helicobacter pylori* infection. However, the protective mechanism of Magnolia officinalis on ethanol-induced gastric injury is still unclear. In this study, UPLC-Q-Exactive Orbitrap-MS was used to analyze the chemical constituents of ethanol extract of Magnolia officinalis, and the protective effects of MOE on ethanol-induced gastric injury in rats were studied from the aspects of oxidative stress and anti-inflammatory, so as to provide experimental basis for the clinical use of Magnolia officinalis in the treatment of gastric ulcer (Figure 8).

Ethanol-induced gastric ulcer is one of the commonly used models to study whether medicines have protective effects on the gastric mucosa. Ethanol directly damages the gastric mucosa and causes gastric mucosal erosion, depletion of bicarbonate, bleeding, and excessive free radicals [32, 33]. In previous studies, Lee et al. indicated that Magnolia officinalis extract had a wide range of inhibitory effect against *H. pylori* growth and reduced mucosal inflammation and epithelial damages in the stomach of the *H. pylori*-infected mice [34]. Our previous studies also found that magnolol reduced nitric oxide content and increased serotonin content, promoted gastrointestinal motility, and alleviated L-arginine-induced gastrointestinal motility disorder in rats [35]. Nitric oxide is involved in both gastrointestinal mucosal defense and injury [15]. They both suggested that Magnolia officinalis has a potential therapeutic effect on gastrointestinal diseases. In the present study, we showed that MOE could decrease gastric mucosa damage in ethanol-induced ulceration models, as evidenced by macroscopic assessment of ulcer lesions and pathologic evaluation. It was proved that the MOE has therapeutic effect on gastric ulcer caused by ethanol in a dose-dependent manner.

Oxidative stress and antioxidant deficiency were considered to be key steps in the development of gastric ulcer [36]. SOD and GSH-Px are important enzymes for scavenging oxygen free radicals in vivo. Instead, MDA is a product of lipid peroxidation, which indirectly reflects the ability of metabolism to scavenge free radicals. These indices are commonly used to measure oxidative damage in the body [37–39]. PGE2 is considered a gastric protective factor by regulating gastric pH, mucus secretion and maintaining the integrity of gastric mucosa. Ethanol-induced oxidative damage can convert prostaglandins into oxidative products, thus inhibiting PGE2 levels in the gastric mucosa [40]. After administration of ethanol in rats, oxidative stress was activated and gastric mucosal cells produced a large amount of oxygen free radicals, which led to gastric mucosal injury and ulcer formation [41]. Magnolol alleviated depression in mice by inhibiting neuroinflammation and oxidative stress in the prefrontal cortex [42]. Honokiol also enhanced both the nonenzymatic and enzymatic antioxidant defense systems, suggested its potential as a natural antioxidant [43]. In this study, MOE has an antioxidant activity in *vitro* and could significantly reduce MDA and increase SOD, GSH-Px, and PGE2 of model rats in *vivo*; these results suggested that MOE had protected the stomach by reducing the proliferation of neutrophils and lipid peroxidation induced by oxidative stress through the antioxidant system.

Inflammation is another important mechanism in the development of gastric ulcer. Gastric ulcer is caused by tissue necrosis caused by gastric mucosal congestion. Immune cells (such as leukocytes and macrophages) engulf necrotic tissues and release pro-inflammatory cytokines (such as TNF-*α*, IL-1*β*, and IL-6) to activate local endothelial cells and epithelial cells [7]. TNF-*α* stimulates neutrophil infiltration, promotes IL-1*β* production and epithelial cell apoptosis, inhibits the recovery of microcirculation around ulcer, and delays ulcer healing. Excessive secretion of IL-6 can activate neutrophils to form inflammatory sites, thus activating oxidative stress and lysosomal enzymes, leading to gastric mucosal damage and gastric ulcer [38, 44]. Magnolol and honokiol, the main components of Magnolia officinalis, have been reported to have anti-inflammatory effects. It has been proved that honokiol has a significant antinociceptive effect on the inflammatory pain model by inhibiting inflammatory factors [45]. In addition, magnolol restrained the expression of TNF-*α*, IL-1*β*, and IL-12 via the regulation of NF-*κ*B and peroxisome proliferator-activated receptor-gamma (PPAR-gamma) pathways and played protective effects on DSS-induced colitis [46]. The results of this study showed that MOE could inhibit the levels of proinflammatory cytokines (TNF-*α*, IL-1*β*, IL-6) induced by ethanol, suggesting that MOE has an anti-inflammatory effect on ethanol-induced gastric ulcer.

The NF-*κ*B signaling pathway is involved in the occurrence of inflammation, is a classic inflammatory pathway, and is related to the process of gastric mucosal injury [2]. The NF-*κ*B family is composed of p65 and p50 subunits; it binds to I*κ*B*α* and forms a trimer in the cytoplasm, which could not play the role of transcriptional regulation [47]. When stimulated by external stimuli (such as proinflammatory cytokines), I*κ*B*α* phosphorylation is induced, and then subunit phosphorylation is induced. NF-*κ*B dimmers are free to translocate to the nucleus and activate target genes, including those that encode induced COX-2 and inflammatory cytokines [7, 48]. COX-2 is an inducible enzyme, which is induced by various stimulating factors, thus promoting the synthesis of prostaglandins and participating in inflammation, pain, and other reactions. It has been reported that the high expression of COX-2 in gastric ulcer tissue is closely related to the healing and recurrence of ulcer. Inhibition of COX-2 can reduce gastric acid secretion, promote the healing of gastric ulcer, and reduce recurrence [49, 50]. In this study, we found that MOE could inhibit the phosphorylation of I*κ*B*α* and NF-*κ*B p65 after ethanol stimulation, and the expression of COX-2 was also significantly downregulated. Combined with the changes of downstream inflammatory factors, MOE could reduce inflammatory response by inhibiting the NF-*κ*B pathway, thus improving gastric tissue injury.

## 5. Conclusion

In conclusion, the chemical constituents in the ethanol extract of Magnolia officinalis are preliminarily identified. MOE has a protective effect on acute gastric injury induced by ethanol in the dose range of 30~120 mg/kg, and the minimum effective dose is 30 mg/kg. It is further confirmed that MOE exerts its effect by inhibiting oxidative stress and the NF-*κ*B inflammatory signaling pathway. Therefore, MOE has further development value for the treatment of gastric ulcer.

## Figures and Tables

**Table 1:** Chemical components of MOE.

No.	Name	RT (min)	Formula	Precursor type	*m*/*z*	Characteristic fragment ions		
1	Magnoside B	2.2	C35H46O20	[M-H]-	785.251	623.2238	477.661	161.0238
2	Syringin	3.03	C17 H24 O9	[M+H]+	390.182	211.0966	193.0861	161.0598
3	Magnoflorine	3.73	C19H24NO3+	[M+H]+	314.1747	298.1074	269.1176	237.0917
4	Lotusine	3.9	C20H24NO4+	[M+H]+	314.1747	269.1171	254.0904	237.0912
5	Magnoside A	4.38	C29H36O15	[M-H]-	623.199	461.1663	315.1094	161.0239
6	Magnoflorine	4.59	C20H24NO4	[M+H]+	342.1705	297.1122	282.0888	265.086
7	Magnoloside E	5.09	C28H34O15	[M-H]-	609.1818	447.1556	161.0238	
8	Magnoloside A	5.26	C29H36O15	[M-H]-	623.1989	461.1664	161.0239	133.0288
9	(R)-oblongine	5.27	C19H24NO3+	[M+H]+	314.1747	282.1494	269.1174	254.0938
10	Magnoloside M	5.63	C29H36O15	[M+H]+	623.1966	461.1655	161.0237	133.029
11	Asimilobine	6.08	C17H17O2N	[M+H]+	268.1328	251.1068	236.0834	219.0806
12	Magnolignan B	7.13	C18H20O5	[M-H]-	315.1253	267.1029	249.0921	239.1089
13	Anonaine	7.96	C17H15O2N	[M+H]+	266.1184	249.0912	219.0807	191.0856
14	Magnolignan E	9.33	C18H18O4	[M-H]-	297.113	267.1025	249.0936	195.2556
15	Randaiol	9.78	C15H14O3	[M-H]-	241.0871	223.0765	213.0921	197.0968
16	Randainol	10.47	C18H18O3	[M-H]-	281.1183	263.1078	245.097	133.065
17	Magnaldehyde B	10.68	C18H16O3	[M-H]-	279.103	263.0717	251.0714	238.0636
18	Magnaldehyde D	11.08	C16H14O3	[M-H]-	253.0872	235.0765	207.0817	
19	Randainal	11.62	C18H16O3	[M-H]-	279.1027	261.0921	233.0969	
20	Obovaaldehyde	11.78	C16H14O4	[M-H]-	269.0823	152.0109	124.0159	
21	Obovatal	12.11	C18H16O4	[M-H]-	295.0976	178.0266		
22	N-Acetylanonaine	10.81	C19H17O3N	[M+H]+	308.1282	249.0911	238.9172	219.0808
23	Honokiol	13.91	C18H18O2	[M-H]-	265.1231	224.0844		
24	Obovatol	14.82	C18H18O3	[M-H]-	281.1187	273.588	164.0474	133.0651
25	Nootkatone	14.9	C15 H22 O	[M+H]+	219.1743	201.1639	145.1014	81.0707
26	Magnolol	14.97	C18H18O2	[M-H]-	265.1234	247.1127	223.0782	
27	Prespatane	16.83	C15 H24	[M+H]+	205.1952	149.1326	109.1017	95.0861
28	(-)-Caryophyllene oxide	17.47	C15 H24 O	[M+H-H2O]+	221.1902	203.1797	147.117	109.1018
29	Piperitylmanolol	29.34	C28H34O2	[M-H]-	401.2488	331.1703	313.1604	247.1126
30	1,2,3,4-Tetramethyl-1,3-cyclopentadiene	30.95	C9 H14	[M-H]-	123.1169	95.0862	81.0707	

## Data Availability

All data used to support the findings of this study are available from the corresponding author upon request.

## Abbreviations

MOE: Ethanol extracts of Magnolia officinalis
H&E: Hematoxylin-eosin
PAS: Periodic acid-Schiff
SOD: Superoxide dismutase
GSH-Px: Glutathione peroxidase
MDA: Malondialdehyde
PGE2: Prostaglandin E2
NF-*κ*B: Nuclear factor kappa B
TNF-*α*: Tumor necrosis factor-alpha
IL-1*β*: Interleukin-1*β*
IL-6: Interleukin-6
COX-2: Cyclooxygenase-2
ELISA: Enzyme-linked immunosorbent assay
OME: Omeprazole
H: Hemorrhage
N: Necrosis
O: Edema
I: Inflammatory cells infiltration in sub-mucosa
mu: Intact mucosal layer
xu: Focal exulceration
gp: Glycoprotein.
